# Nitrogen mustard hydrochloride-induced acute respiratory failure and myelosuppression: A case report

**DOI:** 10.3892/etm.2015.2664

**Published:** 2015-07-29

**Authors:** XIAOJUAN ZHANG, ZHIDAN ZHANG, SONG CHEN, DONGMEI ZHAO, FANGXIAO ZHANG, ZIWEI HU, FENG XIAO, XIAOCHUN MA

**Affiliations:** Intensive Care Unit, The First Affiliated Hospital of China Medical University, Shenyang, Liaoning 110001, P.R. China

**Keywords:** nitrogen mustard hydrochloride, respiratory failure, myelosuppression

## Abstract

Nitrogen mustards are chemical agents that are similar to sulfur mustards, with similar toxicities. The present study describes a case of nitrogen mustard-induced acute respiratory failure and myelosuppression in a 33-year-old man. The patient, who was accidentally exposed to nitrogen mustard hydrochloride in a pharmaceutical factory, exhibited severe inhalation injury and respiratory symptoms. Laboratory tests revealed reduced white blood cell counts and lowered platelet levels during the first 6 days after the skin exposure to nitrogen mustard. Following treatment with mechanical ventilation, immunity-enhancing agents and nutritional supplements for 1 month, the patient successfully recovered and was released from hospital.

## Introduction

Nitrogen mustards (HN-1, HN-2 and HN-3) are chemical agents analogous to sulfur mustards, with similar toxicities. Although nitrogen mustards were applied as chemical warfare agents in the 1930s, they are usually used to treat vitiligo, due to their ability to increase the number of melanosomes within melanocytes (1); however, nitrogen mustard exposure increases the occurrence of tumors (2,3). Nitrogen mustards are toxic to various tissues due to the formation of reactive molecular species. One of the major side effects of these chemicals is severe respiratory toxicity. Symptoms of acute toxicity include chest tightness, a hacking cough and rhinorrhea. The disorders caused by chronic toxicity include bronchiolitis, emphysema and lung fibrosis. The severe symptoms caused by nitrogen mustards are critical factors determining the mortality and long-term survival of patients (4). Nitrogen mustards cause difunctional alkylation through the cross-linking of DNA; therefore, nitrogen mustards can lead to DNA damage, which results in dysfunctional cellular activities, including apoptosis and autophagy (5,6). Nitrogen mustards can, therefore, be lethal upon absorption into the human body, particularly through dermal, respiratory, gastrointestinal and ocular routes of exposure (7). There is no known antidote specifically for toxicities caused by nitrogen mustard, and successfully treated cases are rarely reported. In the present study, a case of nitrogen mustard-induced acute respiratory failure and myelosuppression in a 33-year-old man is described.

## Case report

**Figure 1.**
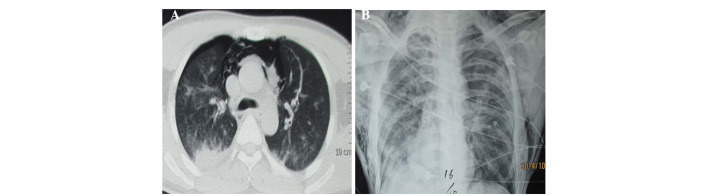
(A) Chest computed tomography scan image of the patient on day 1. The scan shows mediastinal emphysema and pneumothorax. (B) Chest X-ray image of the patient on day 2 following ventilation in the intensive care unit. The image shows pneumothorax and subcutaneous emphysema.

**Figure 2.**
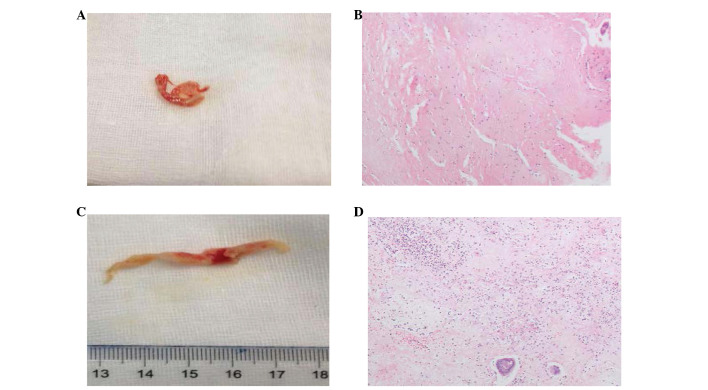
(A and C) Necrotic mucus removed by bronchoscopy. (B and D) Mucosal histology shows the necrosis of the mucus (hematoxylin and eosin staining; magnification, ×10).

**Figure 3.**
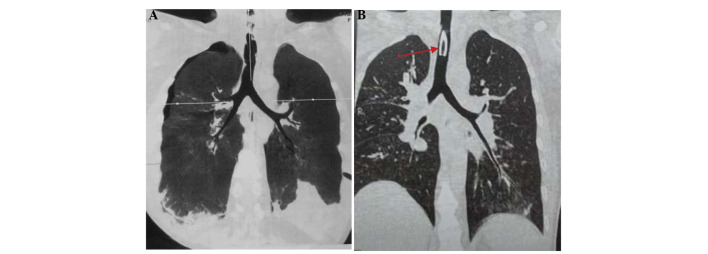
(A) Tracheal 3D CT scan images of the patient on day 13 after treatment showing airway mucosal damage and pneumothorax. (B) Tracheal 3D CT scan images of the patient on day 33 after treatment. Red arrows indicate metal trachea cannula. 3D CT, three-dimensional computed tomography.

**Figure 4.**
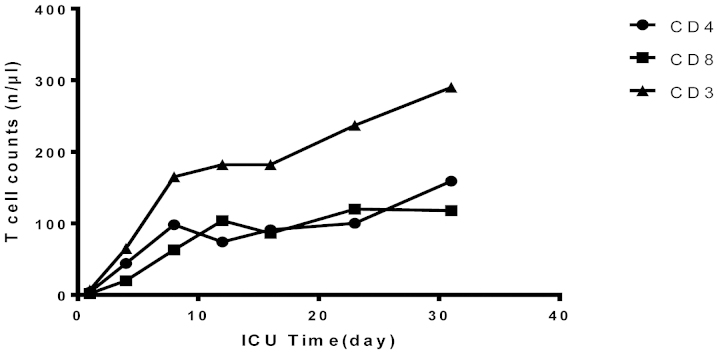
Functional tests of cellular immunity (CD4, CD8 and CD3 levels). Immunity decreased significantly to nearly zero in the first 24 h. Following the application of thymopeptides (1.6 mg/d), immune function recovered slowly, and the levels were up to half of normal levels in a month. ICU, intensive care unit; CD, cluster of differentiation.

**Figure 5.**
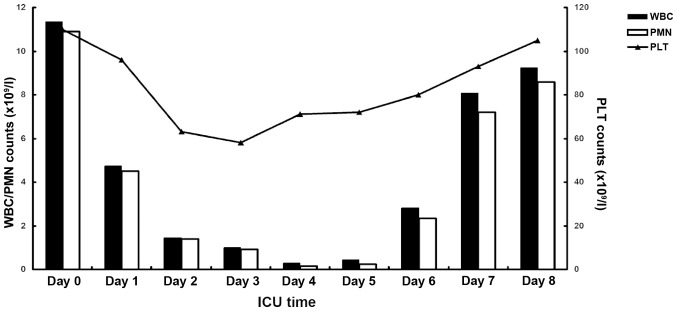
Numbers of WBCs were reduced in the ﬁrst 6 days, largely resulting from the reduction of neutrophils (PMN). The PLT counts were also lower in the first week. Following granulocyte-macrophage colony-stimulating factor administration at 200 mg/day on the second day, the WBC and the PLT counts returned to normal. WBC, white blood cell; PLT, platelet; PMN, polymorphonuclear leukocytes; ICU, intensive care unit.

A 33-year-old man was accidentally exposed to nitrogen mustard hydrochloride in a pharmaceutical factory. The patient experienced progressing dyspnea and was immediately sent to an emergency room at the First Affiliated Hospital of China Medical University (Shenyang, China). Physical examination of the patient showed that his blood pressure was 160/90 mmHg; his respiration rate was 30 breaths/min and his heart rate was 150 bpm. Congestion of the bulbar conjunctiva was observed in the eyes, and moist rales were heard in the lungs. An arterial blood gas test revealed a pH of 7.33, a partial pressure of O_2_ (pO_2_) of 63 mmHg, a pCO_2_ of 51 mmHg and an elevated arterial lactate of 1.9 mmol/l. Blood cell counts revealed significant leukocytosis with a white blood cell count of 11.36×10^9^/l, of which 96% were neutrophils. A chest computed tomography scan showed mediastinal emphysema and pneumothorax (Fig. 1A). The patient had a fever, with a temperature of >39°C, and he was given an oxygen mask. The patient's blood pressure continued to increase to 165/100 mmHg, his heart rate increased to 140–150 bpm and oxygen saturation was 76% in atmospheric air. Re-examination of the arterial blood gas revealed a pH of 7.44, pO_2_ of 49 mmHg and pCO_2_ of 72 mmHg. The patient then underwent tracheotomy and mechanical ventilation. Due to the higher pressure of the mechanical ventilation, the patient suffered the complication of pneumothorax and subcutaneous emphysema (Fig. 1B). Bronchoscopy revealed mucosal edema and necrosis in the airway resulting in a partial obstruction. Mucosal pathology confirmed necrosis (Fig. 2). After 13 days of treatment, the respiratory function of the patient improved markedly, although the patient had developed airway stenosis due to the tracheotomy and mechanical ventilation. The airway stenosis was successfully relieved by local treatment, which included bronchoscopy for sputum suction, and inhalation treatment with mucosalvan (60 mg/d) and budenoside suspension (2 mg/d) (Fig. 3). Despite the improved pulmonary function, the patient had a deteriorated immune function. In the first 24 h, the levels of cellular immune biomarkers [cluster of differentiation 4 (CD4), CD8 and CD3] decreased significantly. The patient was then given thymopeptides (s.c., 1.6 mg/d) to restore immune function; however, this approach was only marginally successful during the acute phase (Fig. 4). The white blood cell count of the patient was decreased in the ﬁrst 6 days, largely resulting from the loss of neutrophils, and the platelet count was also lower than normal (Fig. 5). The patient was given granulocyte-macrophage colony-stimulating factor at 200 mg/day to stimulate neutrophils. Two drops of antibiotics (whole body piperacillin i.v. 4.5 g, levofloxacin eye drops) were administered every 4 h to protect the eyes from nitrogen mustard injury, and potential infection. After being treated for 32 days, the patient fully recovered and was discharged from hospital. Informed consent was obtained from the patient for publishing this case and the associated images.

## Discussion

One of the most severe side effects of nitrogen and sulfur mustard is toxicity to the respiratory tract (4,8). Transdermal injection of nitrogen mustard into rats causes alveolar epithelial cell injury and interalveolar septal thickening (9). These physiological changes cause decreased lung compliance and end-tidal volume, as well as tissue damping and elastance (10). Nitrogen mustard impairs pulmonary function by inducing lung inflammation and oxidative stress (8). Aminoguanidine, a nitric oxide synthase inhibitor, has been reported to alleviate acute lung inflammation and fibrosis caused by nitrogen mustard, suggesting that nitric oxide pathways may play a critical role in nitrogen mustard-induced acute lung injury (11). Nitrogen mustard also increases levels of connective tissue growth factor and matrix metalloproteinase-9 in rats (10). Osterlund *et al* (12) have shown that the activation of extracellular signal-regulated kinase 1/2, p38 mitogen-activated protein kinases and nuclear factor-κB are involved in nitrogen mustard-induced injury to human lung epithelial cells *in vitro*. Activation of these pathways causes subsequent elevations of inflammatory mediators, including tumor necrosis factor-α and intercellular adhesion molecule-1. Ucar *et al* (13) reported that melatonin, an antioxidant molecule and peroxynitrite scavenger, could reduce nitrogen mustard-induced toxicity in the lungs by restoration of oxidative and nitrosative stress markers.

Nitrogen mustard has a high affinity for DNA guanine residues and forms adducts and crosslinks with DNA, RNA and proteins; therefore, nitrogen mustard is potentially mutagenic and carcinogenic (14). Overexpression of the glutathione-S-transferase (GST) subfamily member GSTA2 has been found to protect cells against nitrogen mustard-induced cell cycle arrest and apoptosis (15). Recently, Inturi *et al* (16) studied the mechanism of DNA repair following nitrogen mustard-induced double-strand breaks. They demonstrated that homologous recombination repair pathways were critical in DNA repair following nitrogen mustard toxicity, which could be useful in developing novel therapeutic strategies against nitrogen mustard-associated DNA damage. In addition, DNA repair following DNA damage caused by the nitrogen mustard HN-1 depends on the pathway affecting base excision repair, but the repair process associated with HN-2 primarily requires the activation of a nucleotide excision repair pathway, suggesting that HN-1 and HN-2 may induce different types of cellular damage (17).

As there is no known antidote speciﬁcally for toxicity induced by nitrogen mustard, the most efficient treatment to decrease tissue damage is to reduce absorption by using water or potassium permanganate (18). Other methods include organ support therapy, prevention of infection and nutritional support. To the best of our knowledge, there is no case report in recent years regarding human nitrogen mustard exposure. In the present case, the patient presented with symptoms of severe respiratory failure in the ﬁrst 48 h, with a reduced white blood cell count and complications that included pneumothorax and mediastinal emphysema. Therapies including respiratory support treatment, sputum drainage and airway pressure control played a large role in the successful treatment in this case.

When the temperature is >70°C, nitrogen mustard is in the gaseous state; therefore, at this temperature, nitrogen mustard vapor can potentially be inhaled. The nitrogen mustard in the gaseous state can cause necrosis or apoptosis of bronchial epithelial cells (19). Once nitrogen mustards bind to proteins in the human body, it will be slowly released from the tissues or blood, resulting in multiple organ injuries. The myelosuppression was severe in the present case, and it took 7–10 days for the platelets and white blood cells to recover. Hugel *et al* (20) reported the occurrence of sulfur mustard-induced neutrophil apoptosis. The patient in the present case had symptoms of ocular tissue injury following exposure to nitrogen mustards. Antibiotics were administered to protect the eyes from nitrogen mustard injury and the potential infection; the ocular symptoms disappeared on day 16.

In conclusion, nitrogen mustards can cause severe respiratory injury and they can also damage hematopoietic lineages and immune cells. Timely comprehensive treatment is required to minimize the toxicity caused by nitrogen mustards. The present study demonstrated the importance of multidisciplinary treatments in the intensive care unit.
